# A single GABAergic neuron mediates feedback of odor-evoked signals in the mushroom body of larval *Drosophila*

**DOI:** 10.3389/fncir.2014.00035

**Published:** 2014-04-09

**Authors:** Liria M. Masuda-Nakagawa, Kei Ito, Takeshi Awasaki, Cahir J. O'Kane

**Affiliations:** ^1^Department of Genetics, University of CambridgeCambridge, UK; ^2^Institute of Molecular and Cellular Biosciences, The University of TokyoTokyo, Japan

**Keywords:** mushroom body calyx, APL neuron, olfaction, odor discrimination, inhibition

## Abstract

Inhibition has a central role in defining the selectivity of the responses of higher order neurons to sensory stimuli. However, the circuit mechanisms of regulation of these responses by inhibitory neurons are still unclear. In *Drosophila*, the mushroom bodies (MBs) are necessary for olfactory memory, and by implication for the selectivity of learned responses to specific odors. To understand the circuitry of inhibition in the calyx (the input dendritic region) of the MBs, and its relationship with MB excitatory activity, we used the simple anatomy of the *Drosophila* larval olfactory system to identify any inhibitory inputs that could contribute to the selectivity of MB odor responses. We found that a single neuron accounts for all detectable GABA innervation in the calyx of the MBs, and that this neuron has pre-synaptic terminals in the calyx and post-synaptic branches in the MB lobes (output axonal area). We call this neuron the larval anterior paired lateral (APL) neuron, because of its similarity to the previously described adult APL neuron. Reconstitution of GFP partners (GRASP) suggests that the larval APL makes extensive contacts with the MB intrinsic neurons, Kenyon Cells (KCs), but few contacts with incoming projection neurons (PNs). Using calcium imaging of neuronal activity in live larvae, we show that the larval APL responds to odors, in a mannner that requires output from KCs. Our data suggest that the larval APL is the sole GABAergic neuron that innervates the MB input region and carries inhibitory feedback from the MB output region, consistent with a role in modulating the olfactory selectivity of MB neurons.

## Introduction

In sensory systems, inhibition increases the discriminability of stimuli, by making neuronal responses more selective. Lateral inhibition is one of the major mechanisms that regulate signal input at the early stages of sensory processing; however, the inhibitory circuit mechanisms that play a role in central sensory pathways are less clear. It is now well established that excitatory neurons in sensory cortical areas respond selectively and sparsely to sensory stimuli (Vinje and Gallant, 2000; Hromádka et al., 2008), and this property is considered advantageous for higher order functions for reasons including storage capacity, ease of reading, and energy efficiency (Olshausen and Field, 2004).

The mushroom bodies (MBs) of the insect brain are second-order olfactory centers necessary for odor memory (Davis, 2011). Odor-encoding channels defined by specific olfactory receptors (ORs) project as parallel pathways to the first olfactory center in the brain, the antennal lobe (AL), which corresponds to the olfactory bulb (OB) in mammals, and from there parallel coding is carried further by projection neurons (PNs) to the MBs. At these early stages of the pathway, individual neurons mostly show low selectivity for specific odors, and individual odors are represented by activity in multiple parallel channels; however by contrast, MB neurons Kenyon cells (KCs) respond to odors with high selectivity and sparseness (Perez-Orive et al., 2002; Wang et al., 2004), similarly to mammalian olfactory cortical neurons (Poo and Isacson, 2009). This high selectivity of odor responses is also detected in high levels of odor discrimination in learned behavioral responses, that are dependent on the MBs, and significantly higher than odor discrimination in innate responses that do not depend on the MBs (Parnas et al., 2013).

What are the circuit mechanisms of sparseness and selectivity of odor representations in the central brain? Inhibition is a key component in shaping the transient responses of auditory cortical neurons (Wehr and Zador, 2003) and inhibitory feedforward models have been proposed in this process (Anderson et al., 2000; Priebe and Ferster, 2008). On the other hand, anatomical studies have identified potential feedback neurons in insects including moth (Homberg et al., 1987) and honeybee (Grünewald, 1999a,b), linking the output regions (lobes) and input regions (calyces) of the MBs, and suggesting the presence of a recurrent inhibitory pathway. Recently, a GABAergic Giant Calycal Neuron (GGN) in locust was found to be a normalizing neuron for KC activity, being depolarized by KC activity, and in turn inhibiting odor-induced KC activity (Papadopoulou et al., 2011); hence the GGN should in principle respond to any odor and inhibit any odor-selective response. In adult *Drosophila*, the GABAergic anterior paired lateral (APL) neuron, defined by the GAL4 lines *GH146-GAL4* (Liu and Davis, 2009) and *NP2631-GAL4* (Tanaka et al., 2008; Pitman et al., 2011) innervates the calyx and lobes of the MBs, and could potentially be homologous to the aforementioned neurons from other insects. The APL neuron suppresses olfactory learning in classical olfactory conditioning (Liu and Davis, 2009) and facilitates reversal olfactory learning (Wu et al., 2012), providing evidence of a role of GABAergic innervation in memory acquisition or retrieval. However, the physiological mechanisms of GABAergic innervation in the MBs remain obscure, because of the lack of a defined circuitry to test and interpret the behavioral and physiological data.

The olfactory system of larval *Drosophila* is particularly suited for circuit analysis due to lack of cellular redundancy, and a well characterized olfactory system at a single-cell resolution (Ramaekers et al., 2005; Masuda-Nakagawa et al., 2009). We have shown previously that the calyx of the larval MBs is organized in approximately 34 glomeruli, and that single KCs innervate approximately six different calyx glomeruli, suggesting a combinatorial coding mechanism for odor representation (Masuda-Nakagawa et al., 2005). This is consistent with physiological findings that show a transformation of odor coding along the insect olfactory pathway, from broad representations by PNs, which are the pre-synaptic neurons to KCs, to selective and sparse representations by KCs (Perez-Orive et al., 2002; Wang et al., 2004).

Here we set out to identify all the inhibitory neurons that could regulate excitatory activity within the defined circuitry of the calyx. We found only a single inhibitory neuron that innervates the larval MB calyx pre-synaptically and the MB lobes post-synaptically, and that we designate as the larval APL. The pre-synaptic terminals of the larval APL contact KC processes throughout the calyx, consistent with a role for it in inhibiting KCs directly on their dendrites; we also find a much smaller number of contacts of larval APL pre-synaptic termini on PN termini, suggesting also a limited level of inhibition of PN input to the calyx. Consistent with the neuroanatomy, blocking KC output inhibits odor-induced activity in the larval APL neuron. These results suggest that the sole source of GABAergic inhibition that regulates KC selectivity in the calyx is feedback inhibition that is carried from KC output terminals by the larval APL neuron.

## Materials and methods

### *Drosophila* stocks and crosses

All stocks were maintained on cornmeal-yeast-agar medium. Crosses to test *GAL4* expression patterns were carried out at 25°C. Crosses with *shi^ts^* constructs were carried out at room temperature to avoid neuronal inactivation before temperature shifting.

Expression was driven using *NP0732-GAL4* (recovered from screens described by Hayashi et al., 2002 and Masuda-Nakagawa et al., 2010), *NP2631-GAL4* (Tanaka et al., 2008; Pitman et al., 2011), *GH146-GAL4* (Stocker et al., 1997), *MB247-LexA* (*MB247-LexA::VP16*; Pitman et al., 2011), or *GH146-LexA* (*GH146-LexA::GAD*; Lai et al., 2008). In some individuals, *NP0732-GAL4* also drove expression in a subset of KCs; this could possibly depend on the precise stage of larval development, although we have not determined this, and for neuroanatomy simply used larvae that show little or no expression in KCs. Reporter constructs used were *UAS-mCD8::GFP* (*P{UAS-mCD8::GFP*LL6}; Bloomington stock 5130; Lee and Luo, 1999), *UAS-nSyb::GFP* (Ito et al., 1998), *LexAop-mCD8::GFP* (*P{13xLexAop2-mCD8::GFP*attP2}; Bloomington stock 32203), *UAS-GCaMP3* (*P{UAS-GCaMP3.T*attP40}; Bloomington stock 32116; Tian et al., 2009), *LexAop-GAL80* [*8xLexAop2-IVS-GAL80-WPRE(su(Hw)attP5)*; Bloomington stock 32216], and *UAS-DenMark, UAS-syt::GFP* (Bloomington stock 33065; Nicolaï et al., 2010). Neuronal contacts were detected in progeny of a cross of the GFP Reconstitution Among Synaptic Partners (GRASP) stock *UAS-CD4::spGFP1-10*; *LexAop-CD4::spGFP11* (Gordon and Scott, 2009) to flies carrying *NP0732-GAL4* and either *MB247-LexA* or *GH146-LexA*. Specificity of anti-GFP for reconstituted GFP was tested by confirming absence of labeling in progeny of the GRASP stock crossed to either *GAL4* or *LexA* lines alone. MB silencing was performed using *pJFRC104*, carrying *13xLexAop2-IVS-Dm21-Shibirets1-BP (LexAop-shi)* in *VK00005* (Pfeiffer et al., 2012).

Since *NP0732-GAL4* drove expression in a subset of KCs in some individuals, the effect of GAL4 in KCs was inhibited for live imaging with this line by expressing *LexAop-GAL80* under control of *MB247-LexA*. A recombinant second chromosome carrying both *UAS-GCaMP3* and *LexAop-GAL80* was generated by screening male progeny from *UAS-GCaMP3*/*LexAop-GAL80* females for a darker eye color indicative of the presence of two insertions carrying *w*^+^. Putative recombinants were used to establish stocks that were tested for *UAS-GCaMP3* by anti-GFP and by live imaging of activity, and for *LexAop-GAL80* by the ability to mediate inhibition of *GCaMP3* expression by *MB247-LexA* in adult KCs, where *NP0732-GAL4* was strongly expressed.

### Immunomicroscopy

Primary antibodies were: rabbit anti-GABA (1:1000; Sigma A2052), mouse 4F3 anti-Dlg (1:200 of concentrate from DSHB), rat anti-GFP (1:1000; Nacalai 440426, clone GF090R), rabbit anti-octopamine (1:1000; MoBiTec 1003GE), rabbit anti-dsRed (1:1000; Clontech Living Colors®). Secondary antibodies were Molecular Probes® : goat anti-mouse Alexa 647 (1:200; for anti-Dlg), goat anti-rat Alexa 488 (1:200; for anti-GFP), goat anti-rabbit 546 (1:200; for anti-GABA, or dsRed). Confocal micrographs were acquired using either a Zeiss LSM510 microscope with a 40× NA1.2 objective, or a Zeiss LSM710 microscope with a 40× NA1.3 objective. Surface plots and 3D reconstructions were generated using the Fiji implementation of ImageJ (Schindelin et al., 2012; Schneider et al., 2012). Stereo pair images were generated using two 3D projections separated by a rotation of 5°.

### Imaging of activity

Larvae for imaging the effect of KC blockage on APL activity were normally generated by a cross between *NP2631-GAL4; LexAop-shi/TM6B* and *UAS-GCaMP3; MB247-LexA/TM6B* parents, and selecting non-TM6B (non-Tubby) larval progeny. Seven out of 27 calyces imaged also carried *LexAop-GAL80*, on a *UAS-GCaMP3 LexAop-GAL80* recombinant chromosome described above, but this was driven only by *MB247-LexA* and did not affect *GAL4-dependent* expression of UAS-GCaMP3 in the larval APL. Control larvae lacking *LexAop-shi* were generated by a cross between *NP2631-GAL4* and *UAS-GCaMP3; MB247-LexA/TM6B* parents.

Wandering stage 3rd instar larvae were prepared for imaging as described previously (Masuda-Nakagawa et al., 2009). Images were acquired using a CSU22 spinning disc confocal (Yokogawa Electric Corporation) mounted on an Olympus BX50-WI microscope with a UPlanSApo 40×/NA0.95 air objective, and using an Andor iXon+ DU-888E-CO-#BV EM-CCD camera (Andor, Belfast, UK). Control of illumination and acquisition was performed using controllers supplied by Cairn Research (Faversham, UK); control and image acquisition was performed using Micro-Manager (Edelstein et al., 2010). The room was kept at 22°C, and odor responses were measured a number of times at room temperature; resting fluorescence was recorded for 2 s, followed by odor delivery for 2 s, then 2 s recovery. For temperature shift experiments, larvae were mounted on an aluminium slide, with a hole in the center to allow the larval brain to be positioned using transmitted light, and this was in turn mounted on a temperature-controlled glass Tokai HIT (Shizuoka, Japan) Thermo Plate (MATS-55SF). Temperature was raised by using Tokai HIT temperature controller MATS-LH, setting it between 34 and 36°C. Pilot experiments using a probe (Tokai HIT TSU-0125) placed at exactly the position in the HL3 drop where the brain sample is placed, showed that the drop reached 31°C by 2 min after the start of heating, and reached the temperature of the plate within 4–5 min. To maximize the data that could be acquired from the preparation, recording of responses was started at least 2 min after switching on the hotplate, and repeated approximately once per minute for the next 2–3 min. For cooling, the heater was turned off, and a piece of ice of approximately 300μl was placed on the slide on both sides, of the aluminium slide, which lowered the temperature of the preparation to slightly below room temperature within a minute. Larval APL responses recorded at the restrictive temperature were often barely or not detectable, particularly in preparations expressing *shi^ts^* in KCs, and were only analyzed if the preparation showed some recovery of the response after shifting the temperature down. It was also not possible to record from the calyx in every trial because of movement in and out of focus that occurred during temperature shifting. Responses (Δ*F/F* in a region of interest drawn closely around the area of the signal) were normalized to the average of 2–3 responses at room temperature before temperature shift, and statistical comparisons were performed using a non-parametric Mann-Whitney *U*-test in SPSS software. Control data were obtained from 50 temperature shifts performed on 31 calyces (between one and three temperature shifts per calyx) from 27 brains. *Shibire* data were obtained from 40 temperature shifts performed on 27 calyces from 23 brains.

## Results

### A single gabaergic neuron innervates the calyx of the larval MBs

**Figure 1:**
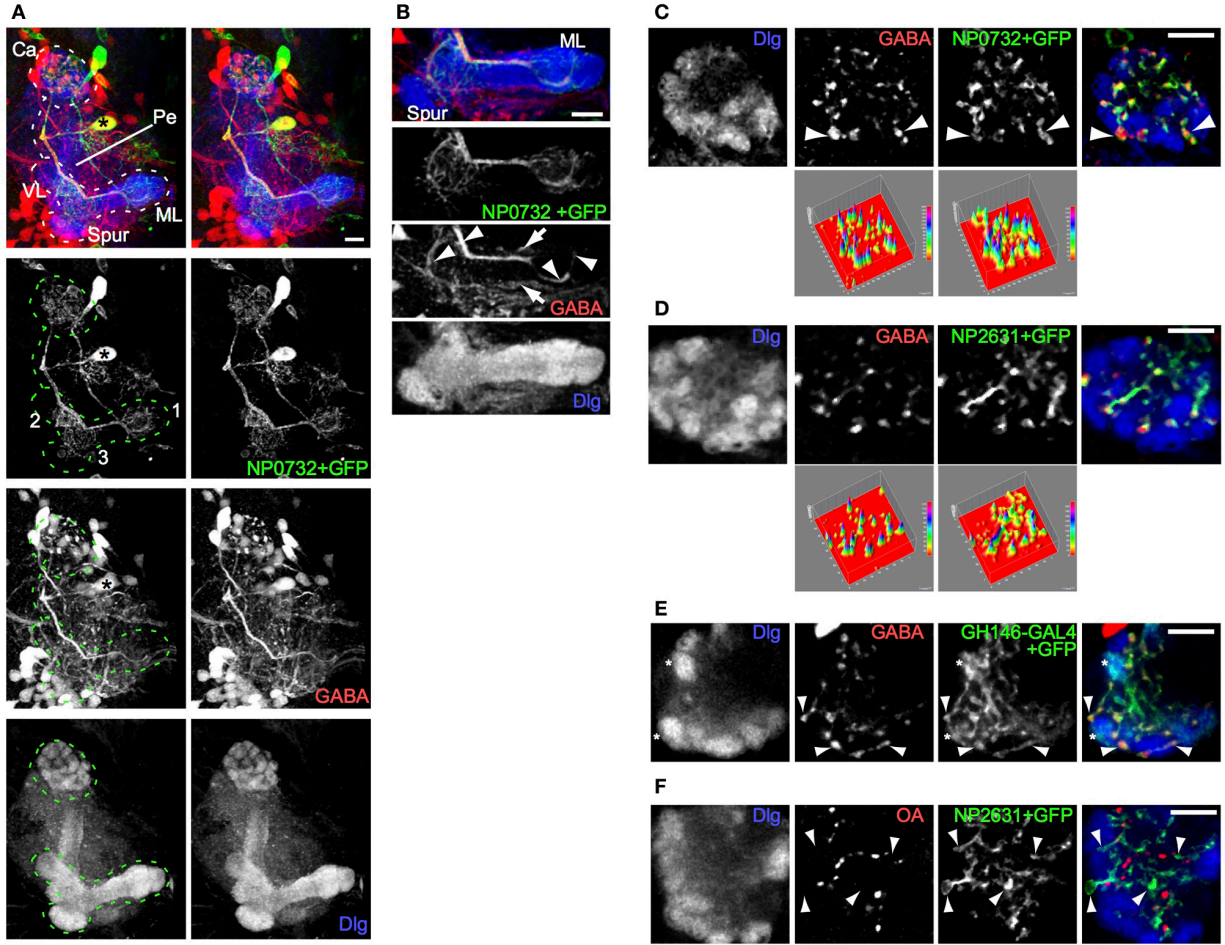
**A single larval GABAergic neuron, resembling the adult APL neuron, innervates the larval calyx. (A)** *NP0732-GAL4* expression, visualized using UAS-mCD8::GFP, is seen in two large cell bodies, in a stereo image of an anterior view of a 3D reconstruction of the larval central brain. One of these (asterisk) posterior to the MB sends a neurite laterally, which branches upwards to the MB calyx (Ca) and downwards to arborizations (labeled in the GFP channel) in (1) the terminal regions of the medial lobe (ML), (2) the proximal region of the vertical lobe (VL), and (3) an area in the region connecting the lower pedunculus (Pe) and vertical lobe. The MB is labeled using anti-Dlg. **(B)** A projection of four confocal sections through the medial lobe of the same preparation. GABA is found in the *NP0732-GAL4* neuron (arrowheads), although weakly and sporadically in its fine arborizations, as well as in one or more neurons innervating the lobes that are not labeled by *NP0732-GAL4* (arrows) **(C)** Confocal section of a larval calyx, showing numerous GABAergic termini, all labeled also with mCD8::GFP expressed under control of *NP0732-GAL4*. Some large GABAergic termini (arrowheads) lie between calyx glomeruli (labeled with anti-Dlg). The two surface plots in the bottom row represent the intensities in the GABA and mCD8::GFP channels; note that virtually all GABA peaks lie within regions of mCD8::GFP expression in the larval APL. **(D)** A similar confocal section and plots of a larval calyx showing overlap of GABAergic termini with mCD8::GFP expressed under control of *NP2631-GAL4*. **(E)** A similar confocal section of a larval calyx showing overlap between GABAergic termini, and axonal and synaptic termini (examples shown with arrowheads) expressing mCD8::GFP under control of *GH146-GAL4*, which is also expressed in a subset of PNs (examples of calyx glomeruli containing these are shown with asterisks). **(F)** Octopaminergic (OA) termini in the calyx do not overlap with termini of the larval APL (examples shown with arrowheads) labeled with mCD8::GFP expressed under control of *NP2631-GAL4*. Scale bars 10μm.

To identify GABAergic neurons that innervate the calyx, we tested a subset of the NP collection of *GAL4* lines (Hayashi et al., 2002; Masuda-Nakagawa et al., 2010), for expression in GABA-expressing neurons that innervate the calyx. One line, *NP0732-GAL4*, expressed in two large neuronal cell bodies as well as about 6 additional small neurons or clusters of neurons per hemisphere (Figure 1A). One of the large cell bodies belonged to a GABAergic neuron, located ventromedially in each brain hemisphere among a few GABAergic cell bodies. It extended one primary process dorsally, that bifurcated at the level of the pedunculus of the MBs, sending one secondary process to innervate the calyx and the other to the MB lobes. In the calyx the secondary process branched into fine processes that terminated in boutons, while toward the lobes a thick secondary process ran along the lower pedunculus and terminated in fine branches in three areas: the terminal regions of the medial lobe, the proximal region of the vertical lobe, and an area in the region connecting the lower pedunculus and vertical lobe (Figure 1A; Movie S1).

Toward the lobes, the main secondary process was rich in GABA; the finer branches were mostly but not entirely free of GABA, and did not show varicosities characteristic of pre-synaptic terminals (Figure 1B). In contrast, both the main and the fine calyx branches were rich in GABA throughout, and contained bouton-like structures strongly immunopositive for GABA (Figures 1A,C). The largest boutons had diameters of a few microns and were located between but not within calyx glomeruli (Figure 1C; Movie S2), where PN boutons synapse with KC dendrites (Masuda-Nakagawa et al., 2005). In the core of the calyx fine processes contained smaller boutons enriched in GABA.

All detectable GABA-containing processes and boutons in the calyx colocalized with CD8::GFP-expressing projections and terminals of the *NP0732-GAL4*-expressing GABAergic neuron (Figure 1C), implying that this is the only GABAergic neuron to innervate widely in the calyx; by contrast, it was not the only GABAergic neuron that innervated the lobes (Figure 1B). The projection pattern of the *NP0732-GAL4*-expressing GABAergic neuron in the MB calyx and lobes resembled that of the adult GABAergic APL neuron, and it is also labeled by *NP2631-GAL4* (Figure 1D) and *GH146-GAL4* (Figure 1E) markers of the adult APL (Tanaka et al., 2008; Liu and Davis, 2009). In contrast to the weak octopamine expression reported in the adult APL (Wu et al., 2013), we could not detect octopamine expression in the larval APL (Figure 1F). Nevertheless, given the strong anatomical and expression similarities with the adult APL, we named this GABAergic neuron as the larval APL neuron, reflecting at least some functional equivalence; we do not know whether the larval and adult APL neurons are developmentally identical, but given the re-use of many larval neurons for similar roles in the adult, this may indeed be the case.

### Pre-synaptic and post-synaptic innervation of the larval APL

**Figure 2:**
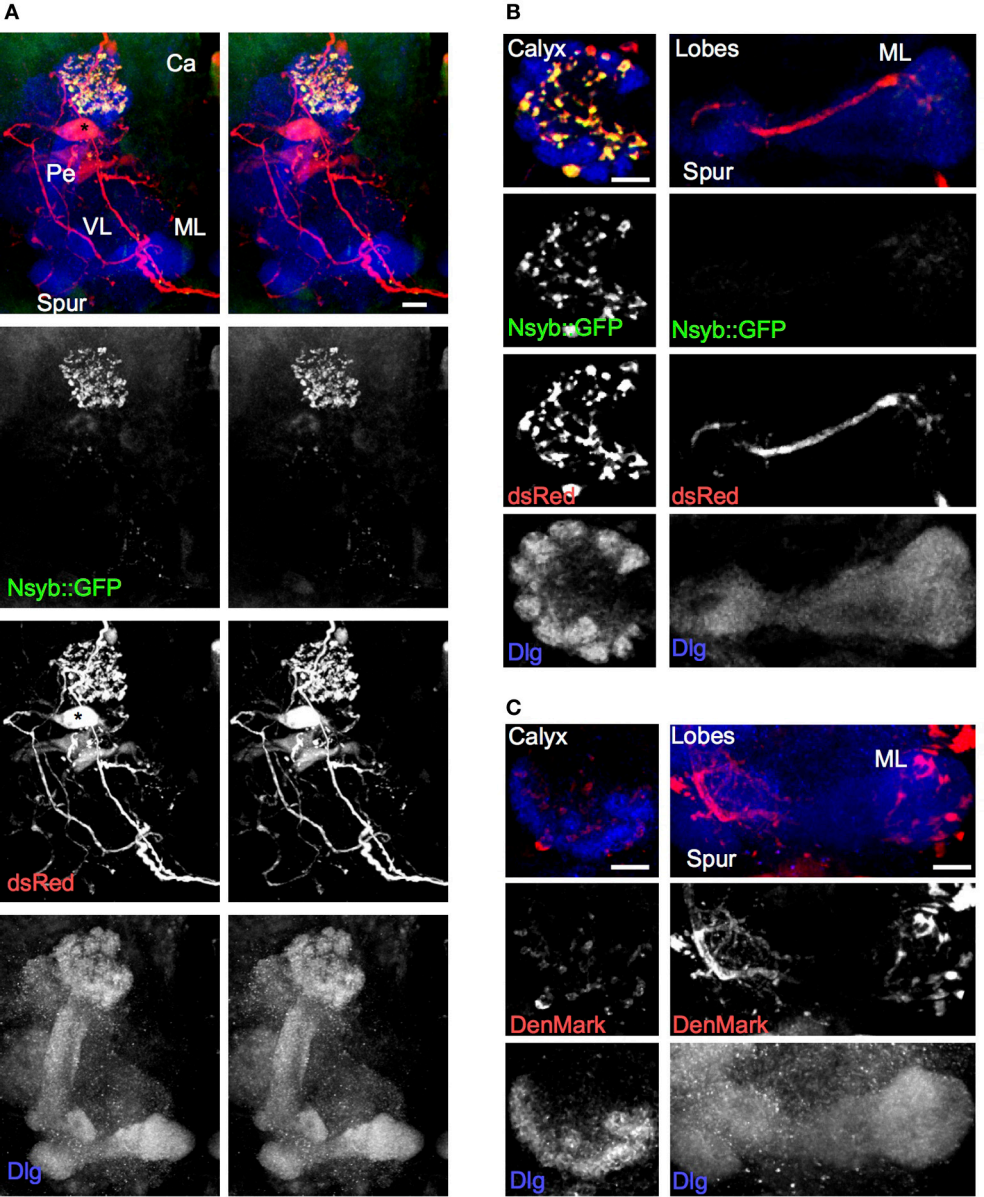
**The larval APL neuron has pre-synaptic termini in the calyx, and termini with dendritic characteristics in both lobes and calyx. (A)** Using *NP0732-GAL4* to express dsRed to label the larval APL cell outline, and nSyb::GFP to label pre-synaptic termini, shows strong labeling of pre-synaptic termini in the calyx, but not in the lobes, spur or pedunculus. **(B)** Sections through a calyx (left column) and medial lobe (right column) of a brain of the same genotype as **(A)**. Pre-synaptic terminals, labeled with nSyb::GFP, are widespread and heavily labeled in the calyx, although low levels of nSyb::GFP are also seen in larval APL terminals in the lobes. **(C)** Using *NP0732-GAL4* to express the dendritic marker DenMark, reveals strong labeling of larval APL projections in the lobes (right column), and weak labeling of the calyx (left column). All views are anterior. Scale bars, 10μm. Abbreviations as in Figure 1.

To understand the directionality of signaling through the larval APL, we examined the localization of pre-synaptic and dendritic markers expressed in it, using *NP0732-GAL4*. The pre-synaptic marker nSyb::GFP localized specifically to larval APL terminals in the calyx, but was absent from most of its projections elsewhere including the MB lobes, as visualized with the cytoplasmic marker dsRed (Figures 2A,B; Movie S3). This suggests that the larval APL terminals in the calyx are pre-synaptic and are sites of GABA release, and that the projections in the MB lobes are overwhelmingly dendritic. The dendritic marker, DenMark (Nicolaï et al., 2010), also strongly labeled the secondary process and fine branches of the larval APL in the vertical and medial lobes (Figure 2C), consistent with these projections being dendritic. DenMark also weakly labeled larval APL terminals in the calyx, suggesting the possibility of some dendritic specializations in them in addition to the pre-synaptic vesicle release machinery identified by nSyb::GFP.

### Grasp between KCs and larval APL

**Figure 3:**
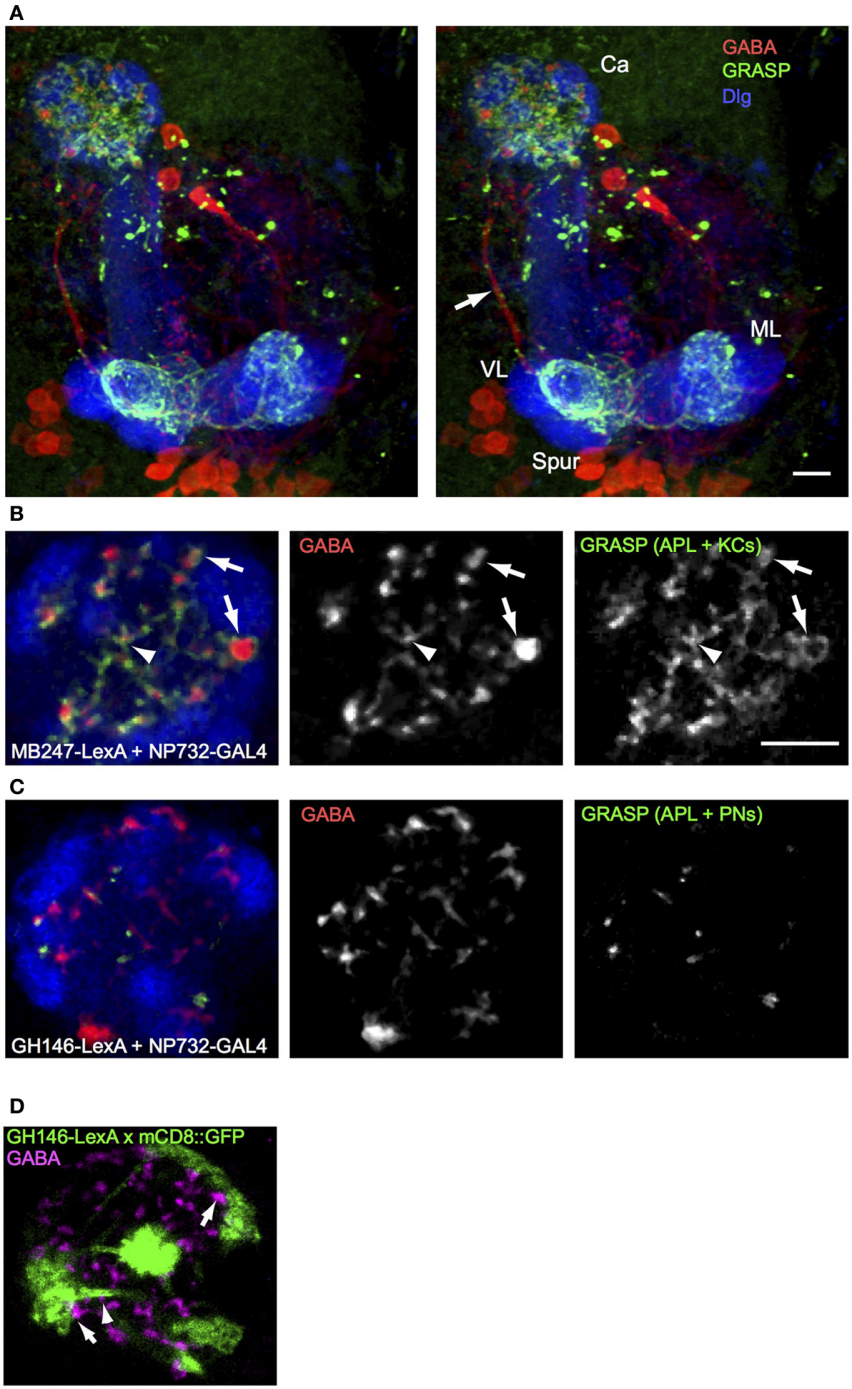
**GRASP shows extensive contacts of larval APL with KCs in MB calyx and lobes, and few contacts with PNs. (A)** An anterior stereo view of a larval brain expressing *MB247-LexA, NP0732-GAL4*, and the two GRASP constructs *UAS-CD4::spGFP1-10* and *LexAop-CD4::spGFP11*, labeled with anti-GABA (red), anti-GFP (green) and anti-Dlg (blue). Reconstituted GFP signal is present only in calyx and lobe projections (labels as in Figures 1, 2) of the larval APL and is absent from the main larval APL neurite (arrow). **(B)** A section through the calyx of a similar larva, showing widespread GRASP signal between larval APL and KCs, both on larger GABA boutons between glomeruli (arrows) and in the interior of the calyx (arrowheads). **(C)** Occasional GRASP signals between larval APL termini, and PNs labeled with *GH146-LexA::GAD*. **(D)** Confocal section of a calyx that expresses mCD8::GFP under control of *GH146-LexA::GAD* (green), labeled with anti-GABA (magenta). GFP expression is found only in PNs, but not in larval APL GABAergic termini. Some GABAergic termini are found adjacent to PN pre-synaptic trees that define calyx glomeruli (arrows), and to PN axons (arrows). Scale bars 10μm, for **(A)**, and **(B–D)**. Abbreviations as in Figure 1.

To understand the connectivity of the larval APL neuron within the calyx we performed GRASP (Gordon and Scott, 2009) between the larval APL and two of its potential synaptic partners in the calyx, KCs and PNs. Using *NP0732-GAL4* and *MB247-LexA* to drive expression of split GFP components in larval APL and KCs respectively, we observed a strong and widespread GRASP signal in both the MB calyx and lobes (Figure 3A; Movie S4). In the calyx, GRASP signal appeared around synaptic boutons of the larval APL, located between calyx glomeruli or at the edges of calyx glomeruli (Figure 3B) where larval APL termini could potentially contact KC dendritic claws that wrap around PN boutons (Masuda-Nakagawa et al., 2005). We also observed GRASP signal around the smaller larval APL boutons in the core of the calyx, indicating that KC dendritic shafts are also likely sites of synaptic contacts between larval APL and KCs. All GABA-positive boutons appeared to be labeled with GFP, suggesting that all larval APL boutons make synaptic contacts with KCs, at locations where they form claws around calyx glomeruli or on their dendritic shafts.

In contrast to the widespread GRASP signal between larval APL and KCs, GRASP between the larval APL and PNs, using *NP0732-GAL4* and *GH146-LexA* respectively, showed a signal on only occasional GABA termini. Only a few dots of GRASP labeling were observed (Figure 3C), located between glomeruli or in the core of the calyx, but not within glomeruli where the pre-synaptic terminal branches of PNs are localized (Masuda-Nakagawa et al., 2005). Consistent with the localization of larval APL-PN GRASP signal, occasional GABA-labeled larval APL boutons could be found close to the axonal projections of PNs within the calyx, or to the surface of the pre-synaptic bouton cluster of individual PNs in calyx glomeruli, but not within glomeruli (Figure 3D). Note that *GH146-LexA* did not show detectable expression in the larval APL (Figure 3D), unlike *GH146-GAL4* in the larval (Figure 1E) or adult APL (e.g., Liu and Davis, 2009).

### The larval APL responds to odors

**Figure 4:**
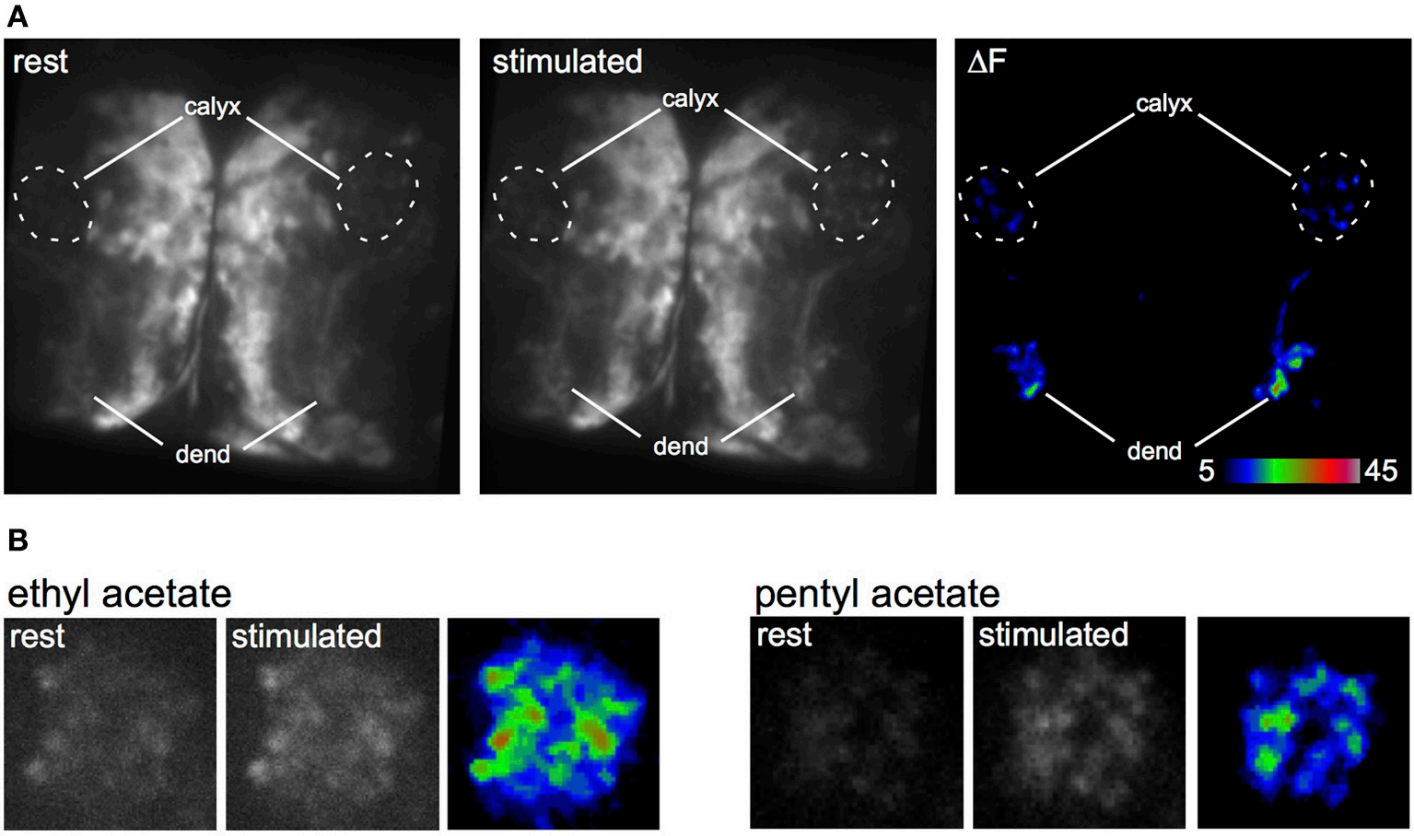
**Odor-evoked responses in the larval APL. (A)** Response of two larval APLs from the same brain to a pulse of ethyl acetate, shown by increased GCaMP fluorescence in both dendrites (dend) and calyx, in a larva that expresses GCaMP3 under control of *NP2631-GAL4*. **(B)** Response of larval APL termini in slightly different planes of the same calyx to ethyl acetate or pentyl acetate, in a larva that expresses GCaMP3 under control of *NP0732-GAL4*. This larva also expresses LexAop-GAL80 driven by *MB247-LexA* as a precaution to prevent expression of GCaMP3 in KCs.

To analyze the response of the larval APL to odors, we expressed the genetically encoded calcium indicator GCaMP3 (Tian et al., 2009) in the larval APL, and recorded calcium responses to odor stimulation. Odor-evoked activity was observed throughout the larval APL (Figure 4A), although we could not distinguish any temporal difference in activity between larval APL dendrites in the MB lobes, and its pre-synaptic terminals in the calyx (Figure 4A and Movies S5, S6). Larval APL activity in the calyx appears to be spread across the entire calyx, even for two different odors that stimulate quite different distributions of olfactory sensory receptors (Kreher et al., 2008) (Figure 4B; Movies S7–S10). Since PN input, and hence odor representations, are spatially localized in the larval calyx (e.g., Masuda-Nakagawa et al., 2009), our data suggest that the larval APL is not selective to odors but acts as a general sensor of odor-induced activity in the MB lobes, that signals this activity level across the entire calyx.

### Odor-evoked activity in larval APL depends on KC output

**Figure 5:**
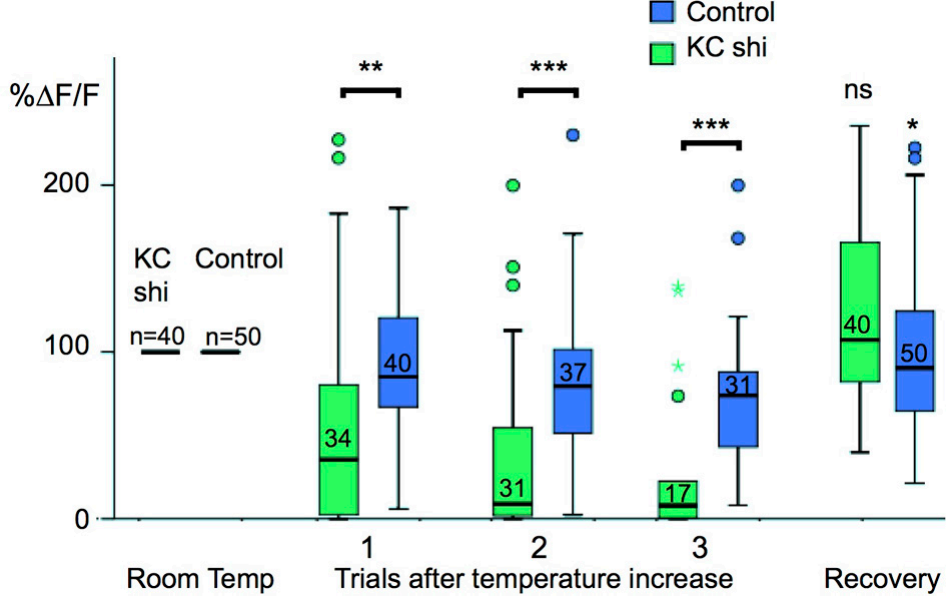
**Odor-evoked responses in the larval APL are dependent on KC input**. Box plots of larval APL responses to ethyl acetate, visualized using *NP2631-GAL4* and *UAS-GCaMP3*, at room temperature, and three successive trials after a shift to restrictive temperature, when KC neurotransmission is blocked by expression of *shibire^ts^* using *MB247-LexA*, (KC shi), or in control larvae that are treated identically but do not express *shibire^ts^*. Each value of Δ*F/F* was normalized to the average response value of the same calyx at room temperature (100%). Shibire-expressing calyces (KC shi) were compared to non-expressing calyces at each time point; responses after returning to room temperature (“Recovery”) were compared to responses of the same calyx prior to temperature shift. Pairwise comparisons were carried out using Mann-Whitney *U*-tests (ns, not significant; ^*^P < 0.05; ^**^P < 0.005; ^***^P < 0.001). Boxes show the second and third quartiles, separated by the median; whiskers extend 1.5× the length of each box, or to the most extreme value, whichever is shorter; outliers are labeled as circles and colored asterisks. A small number of individual outliers lay outside the range shown in the graph, but were included in the statistical analysis. The number of temperature shifts analyzed for each condition is shown just above each median bar.

To understand the functional connectivity of the larval APL in the MB circuitry, we blocked the output activity of KCs by expressing *shibire^ts^* in KCs using *MB247-LexA*, and recorded odor responses in the larval APL neuron using *NP2631-GAL4* and *UAS-GCaMP3*. Each experiment consisted of three cycles of repeated recordings: a first cycle of recordings at 22°C, a second cycle of recordings after raising the temperature to at least 31°C to induce blocking of KC neurotransmission by *shibire^ts^*, and a third cycle of recordings after returning to permissive temperature to check for recovery of the system in case of loss of activity. During blockage of KC neurotransmission at restrictive temperature, larval APL activity gradually decreased with repeated odor stimuli (Figure 5); a gradual decrease is expected since *shi^ts^* blocks endocytosis rather than exocytosis, and therefore gradually depletes the synaptic vesicle pool (e.g., Delgado et al., 2000). This decrease in larval APL activity was significantly larger than that seen under the same conditions in control larvae that lacked the *shi^ts^* transgene; it could also be reversed by recovery at the permissive temperature (Figure 5).

## Discussion

### Polarity and connections of the larval APL

A single GABAergic neuron, the larval APL neuron, accounts for all the GABAergic innervation that we can detect in the larval MB calyx. This neuron is highly polarized, with overwhelmingly dendritic processes in the MB lobes and pre-synaptic processes in the calyx (Figure 2). It makes extensive contacts with KCs in both the calyx and lobes (Figure 3). Its polarity suggests that it receives input from KCs in the MB lobes and releases GABA onto KC dendrites in the calyx. These features strongly support a role for it as a feedback neuron, mediating inhibition of KC depolarization across the whole calyx, in response to KC outputs.

**Figure 6:**
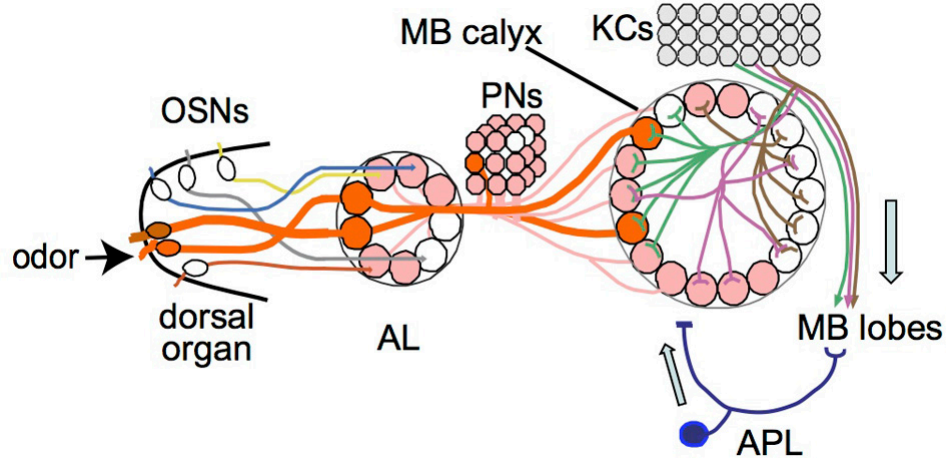
**Model of larval olfactory pathway with feedback inhibition in the mushroom body**. Single olfactory sensory neurons (OSNs) project to single antennal lobe (AL) glomeruli, from where single projection neurons (PNs) project mostly to single glomeruli in the mushroom body (MB) calyx. Odors normally activate multiple OSNs and hence multiple PNs and calyx glomeruli (two examples shown in orange and thick lines). If enough dendritic branches of an individual Kenyon cell (KC) are activated by inputs from multiple calyx glomeruli, the KC transmits signals to the MB lobes. The APL responds to KC activity in the MB lobes and transmits an inhibitory feedback signal (GABA) to the MB input region in the calyx. Adapted from Masuda-Nakagawa et al. (2009).

Imaging of odor-evoked activity in the larval APL further supports its role as a feedback neuron. It responds to at least two different odors, ethyl acetate and pentyl acetate (Figure 4), in both cases with activity throughout the calyx. The odor responses of the larval APL appear to be evoked by KC output. Larval APL activity is inhibited by blocking KC output (Figure 5), and both the polarity of the larval APL, and its extensive contacts with KCs in the MB lobes (Figures 2, 3), suggest direct synaptic transmission from KCs to larval APL dendrites in the lobes. Therefore, the larval APL, by releasing GABA in response to odor-evoked activity in KCs, would mediate negative feedback from KC output to the calyx, in a manner that is neither odor-selective nor KC subset-selective (Figure 6).

While KCs appear to be the main target of the larval APL in the calyx (Figure 3B), there are occasional sites of contact of larval APL terminals with PNs (Figure 3C). Therefore some pre-synaptic inhibition of PN activity could also contribute to a general inhibition of neuronal activity in the calyx. Other synaptic partners of the larval APL in the calyx might include as-yet non-characterized non-PN and non-KC extrinsic neurons.

Some larval APL boutons in the calyx are relatively large (4–5μm diameter) and lie between glomeruli (Figures 1C, 2B). The larval APL, like the adult APL and locust GGN, is presumably non-spiking (Papadopoulou et al., 2011), and so large GABA stores may be required for continual GABA release, as well as to provide additional GABA on olfactory stimulation. The extraglomerular sites of these boutons hint that GABA might diffuse within the calyx and create a general inhibitory environment; the calyx is surrounded by glia but has no obvious internal glial barriers to neurotransmitter diffusion (Leiss et al., 2009; LMM-N, unpublished results). However, the large boutons are surrounded by KCs (Figure 3B), despite their extraglomerular locations. The main barrier to diffusion of GABA in the calyx must therefore be the localization and activity of plasma membrane GABA transporters within it, about which nothing is currently known.

The larval APL might potentially be activated in the calyx by PNs, or from pre-synaptic specializations on KC dendrites (Christiansen et al., 2011). However, we see no evidence for this; PN activity and KC excitatory post-synaptic potentials are expected to show odor-specific localization in the calyx (Masuda-Nakagawa et al., 2009), and we observed only broadly uniform responses of the larval APL terminals across the entire calyx (Figure 4).

### Comparisons of larval and adult APL and feedback neuron architecture

Despite the obvious similarities, there are some major differences between the larval and adult APL neurons. Whereas the larval APL innervates the entire calyx but only certain regions of the lobes, (Figure 1), the adult APL innervates the whole of the calyx, lobes and pedunculus (Liu and Davis, 2009; Wu et al., 2013). Furthermore, the larval APL is strongly polarized, appearing overwhelmingly dendritic in the MB lobes and pre-synaptic in the calyx, albeit with some dendritic label in the calyx and a small amount of pre-synaptic marker in the lobes (Figure 2). By contrast, the adult APL appears completely non-polarized, strongly expressing both pre-synaptic Syt::HA and dendritic DenMark in both the calyx and the lobes (Wu et al., 2013); therefore, in addition to being a feedback neuron, it might be able to mediate local inhibitory circuits within both the calyx and lobes. Other fly neurons that anatomically appear highly polarized can also have mixed axodendritic projections; these include some classes of adult KCs that have pre-synaptic as well as post-synaptic specializations in the calyx (Christiansen et al., 2011), although the post-synaptic targets of these pre-synaptic specializations, and whether the calycal microcircuits that they could mediate include the APL, are unknown.

Octopamine has recently been reported in the adult APL, together with evidence that knocking down its synthesis can impair anesthesia-resistant memory (Wu et al., 2013). However, we cannot detect any specific octopamine immunoreactivity in the larval APL, even when image intensity is saturating in most other octopaminergic termini in the calyx (Figure 1F); this makes it unlikely that we are missing weak immunoreactivity similar to that reported in the adult APL (Wu et al., 2013). Whether this reflects an absence of anesthesia-resistant memory in larvae is unknown.

The larval APL appears broadly similar to GABAergic MB neurons in a number of other insects. The locust GGN (Papadopoulou et al., 2011) has projections in the MB lobes and calyx that resemble that of the *Drosophila* larval APL, and consistent with our findings, it receives monosynaptic inputs from KCs. Like the *Drosophila* larval APL and unlike the adult APL, it shows extensive arborization in restricted areas of the KC lobes (Papadopoulou et al., 2011); and unlike either stage of the *Drosophila* APL, the locust GGN also innervates the lateral horn. There is not yet any molecular evidence concerning GGN axo-dendritic polarity. GABAergic “feedback neurons” that connect subregions of the MB lobes and calyces, and whose processes in the lobes appear to be post-synaptic, are also been seen in other insects including honeybee or the moth *Manduca*, where there are about 50 or 150, respectively (Homberg et al., 1987; Grünewald, 1999a), rather than just one as in *Drosophila*. The honeybee feedback neurons respond to various sensory stimuli via input from the MB lobes, and their activity can be influenced by learning (Grünewald, 1999b; Haehnel and Menzel, 2010, 2012).

Inhibitory feedback neurons therefore appear to be an ancient component of MBs, dating back at least some 300 M years to the divergence of Diptera and Orthoptera. However, the detailed specifications of these neurons differ in properties such as the numbers of neurons, in the regions of the lobes and calyx, and hence in the individual KCs innervated, and in the potential for local inhibitory circuits within the lobes or calyx.

### Role of inhibition in the calyx

KC responses to odors are both sparse, with a high input threshold for firing, and transient, consisting of only around 1–10 spikes (Perez-Orive et al., 2002). This is achieved by the organization of the calyx: KCs possess a dendritic organization that makes them combinatorial integrators of olfactory inputs (Masuda-Nakagawa et al., 2005), and concomitant activation of multiple PN inputs is required to make KCs fire (Gruntman and Turner, 2013; Li et al., 2013); KCs are also the major target of the larval APL inhibitory feedback neuron, which might contribute to the high threshold of firing of KCs. In the adult APL, blocking RDL (GABA_A_) receptor expression in KCs, or reducing GABA synthesis in the APL using *GH146-GAL4*, increased the numbers of KCs firing in response to odors (Lei et al., 2013). The inhibitory feedback provided by the larval APL neuron from KC pre-synapses to KC dendrites is also likely to make KC responses transient.

### Role of APL in learning

In adult *Drosophila*, knockdown of GABA synthesis in the adult APL neuron increases associative olfactory learning, implying that adult APL activity inhibits learning (Liu and Davis, 2009). However in reversal learning using a similar olfactory choice paradigm, but with a second round of training in which the conditioned stimulus is swapped, knockdown of GABA synthesis in the adult APL impairs learning (Wu et al., 2012). How could these observations be explained and reconciled, in light of the inhibitory feedback circuit just discussed? When APL inhibitory activity in the calyx is impaired, KCs would have lower firing thresholds and hence increased sensitivity to conditioned stimuli; associative learning could thus in some circumstances be enhanced (e.g., Liu and Davis, 2009). However, higher sensitivity will lower discrimination, since there will be greater overlap in the populations of KCs responding to different odors. Therefore in other circumstances, impaired ability to discriminate odors could lead to lower learning scores (e.g., Wu et al., 2012).

## Conclusions

The larval calyx is a microcircuit for odor discrimination, receiving olfactory input from PNs to form neural representations in the KCs. We have characterized a key component of the circuit, the larval APL neuron, a feedback inhibitory neuron that innervates the MB lobes post-synaptically and the calyx pre-synaptically. The larval APL neuron has a clear polarity, with dendrites in the MB lobes that can be activated by KC output, and with GABA-containing terminals in the calyx, where KC dendrites are its main post-synaptic targets (Figure 6). It is the only GABAergic neuron that arborizes throughout the calyx, and therefore is the only inhibitory component that can regulate the sparseness of KC olfactory responses, and their transientness, both essential for odor discrimination. Our identification of the larval APL as the sole channel for inhibitory feedback in the larval MB advances our system-level understanding of how specific sensory qualities can be selectively encoded in a memory center in the higher brain.

## Author contributions

Liria M. Masuda-Nakagawa designed the project, performed experiments, analyzed data, and wrote the paper. Kei Ito provided infrastructural support. Takeshi Awasaki and Kei Ito provided unpublished larval expression patterns, Cahir J. O'Kane co-designed the project, generated reagents, analyzed data, co-wrote the paper.

### Conflict of interest statement

The authors declare that the research was conducted in the absence of any commercial or financial relationships that could be construed as a potential conflict of interest.

## Supplementary material

Movie S1 **Y-axis rotation of a confocal reconstruction of the mushroom body preparation used to generate Figure 1A**.

Movie S2 **Y-axis rotation of a confocal reconstruction of the calyx used to generate Figure 1C**.

Movie S3 **Y-axis rotation of a confocal reconstruction of the mushroom body preparation used to generate Figure 2A**.

Movie S4 **Y-axis rotation of a confocal reconstruction of the GRASP preparation used to generate Figure 3A**.

Movie S5 **Activity of GCaMP3 under control of *NP2631-GAL4* in response to a puff of ethyl acetate, from the larval brain shown in Figure 4A**.

Movie S6 **False color of Movie S5**.

Movie S7 **Activity of GCaMP3 under control of *NP0732-GAL4* in response to a puff of ethyl acetate, from the larval calyx shown in Figure 4B**.

Movie S8 **False color of Movie S7**.

Movie S9 **Activity of GCaMP3 under control of *NP0732-GAL4* in response to a puff of pentyl acetate, from the larval calyx shown in Figure 4B**.

Movie S10 **False color of Movie S9**.
